# Effect of the Cigarette Smoke Component, 4-(Methylnitrosamino)-1-(3-Pyridyl)-1-Butanone (NNK), on Physiological and Molecular Parameters of Thiamin Uptake by Pancreatic Acinar Cells

**DOI:** 10.1371/journal.pone.0078853

**Published:** 2013-11-07

**Authors:** Padmanabhan Srinivasan, Veedamali S. Subramanian, Hamid M. Said

**Affiliations:** 1 Department of Medical Research, VA Medical Center, Long Beach, California, United States of America; 2 Departments of Medicine and Physiology/Biophysics, University of California Irvine, Irvine, California, United States of America; University of Florida, United States of America

## Abstract

Thiamin is indispensable for the normal function of pancreatic acinar cells. These cells take up thiamin via specific carrier-mediated process that involves thiamin transporter-1 and -2 (THTR-1 and THTR-2; products of *SLC19A2* and *SLC19A3* genes, respectively). In this study we examined the effect of chronic exposure of pancreatic acinar cells *in vitro* (pancreatic acinar 266-6 cells) and *in vivo* (wild-type and transgenic mice carrying the *SLC19A2* and *SLC19A3* promoters) to the cigarette smoke component 4-(methylnitrosamino)-1-(3-pyridyl)-1-butanone (NNK) on physiological and molecular parameters of the thiamin uptake process. The results show that chronic exposure of 266-6 cells to NNK (3 µM, 24 h) leads to a significant inhibition in thiamin uptake. The inhibition was associated with a significant decrease in the level of expression of THTR-1 and -2 at the protein and mRNA levels as well as in the activity of *SLC19A2* and *SLC19A3* promoters. Similarly chronic exposure of mice to NNK (IP 10 mg/100 g body weight, three times/week for 2 weeks) leads to a significant inhibition in thiamin uptake by freshly isolated pancreatic acinar cells, as well as in the level of expression of THTR-1 and -2 protein and mRNA. Furthermore, activity of the *SLC19A2* and *SLC19A3* promoters expressed in transgenic mice were significantly suppressed by chronic exposure to NNK. The effect of NNK on the activity of the *SLC19A2* and *SLC19A3* promoters was not mediated via changes in their methylation profile, rather it appears to be exerted via an SP1/GG and SP1/GC cis-regulatory elements in these promoters, respectively. These results demonstrate, for the first time, that chronic exposure of pancreatic acinar cells to NNK negatively impacts the physiological and molecular parameters of thiamin uptake by pancreatic acinar cells and that this effect is exerted, at least in part, at the level of transcription of the *SLC19A2* and *SLC19A3* genes.

## Introduction

Thiamin (vitamin B1) is indispensable for the normal function and health of pancreatic cells due to its involvement in oxidative energy (sugar) metabolism and ATP production in the mitochondria [1]–[2] via its role as a cofactor for multiple enzymes (transketolase, pyruvate dehydrogenase, alpha-ketoglutarate dehydrogenase and branched chain ketoacid dehydrogenase). The vitamin also plays an important role in reducing cellular oxidative stress via maintaining normal cellular redox state [3], [4] and in normal mitochondrial physiology [5]. Thus, low intracellular levels of thiamin leads to impairment in oxidative energy metabolism (acute energy failure) and to a propensity for oxidative stress [2]–[6]; it also leads to impairment in the structure and function of mitochondria [5].

The pancreas is an important organ of the digestive system, and diseases of this organ lead to significant morbidity and mortality. Thiamin deficiency negatively impacts both the exocrine (severe reduction in its content of digestive enzymes; [7]) and endocrine (impairment in insulin secretion; [8], [9]) functions of the pancreas. The latter is exemplified by the development of diabetes in patients with the autosomal recessive disorder thiamin responsive megaloblastic anemia (TRMA), which is caused by mutations in thiamin transporter -1, the product of the *SLC19A2* gene [10], [11].

Pancreatic cells maintain high level of thiamin [12], despite their inability for *de novo* synthesis of the vitamin. These cells obtain thiamin from their surrounding (circulation) via transport across cell membranes. We have recently characterized the mechanism of thiamin uptake by pancreatic acinar (as well as beta/islets) cells and showed the involvement of specific and regulated carrier-mediated process [13], [14]. Using *SLC19A2* and *SLC19A3* knockout mouse models, we also showed that both of the thiamin transporters -1 and -2 (THTR-1 and THTR-2) are involved in thiamin uptake by pancreatic acinar cells [15]. Little is currently known about the effect of common environmental factors that are known to negatively impact pancreatic physiology and health on the thiamin uptake process of pancreatic acinar cells. One such factor is cigarette smoke (and its constituents), where a wealth of epidemiological and experimental studies has linked this environmental factor to the development and progression of pancreatic injury/disease (e. g., chronic and acute pancreatitis, pancreatic cancer) [16]–[22]. Indeed CS induces marked functional and pathological changes in the exocrine pancreas [23]–[25], and these adverse effects can also be seen with the specific cigarette smoke constituent 4-(methylnitrosamino)-1-(3-pyridyl)-1-butanone (NNK) [23], [26]. NNK is a compound that is formed during tobacco curing process [27], and predominantly accumulates in the pancreas [28]. The mechanism(s) by which chronic exposure to cigarette smoke negatively affects cell physiology are not fully understood but appears to be multi-factorial and include alterations in gene expression (with both inhibition and induction being reported; [29]–[31]; also see micro-array data link http://www.ebi.ac.uk/gxa/qrs?gprop_0=&gnot_0=&gval_0=slc&fact_1=&fexp_1=UP_DOWN&fmex_1=&fval_1=smoking&view=list&searchMode=simple), oxidative stress [20], and mitochondrial dysfunction [32]. Negative effect(s) of cigarette smoke or its constituents on pancreatic physiology of thiamin (a vitamin whose cellular deficiency also leads to oxidative stress, mitochondrial dysfunction, and other metabolic disturbances; 2–6) could also contribute to the deleterious effect of these external factors on the physiology and health of the pancreas. Our aim in this study was to examine the effect of chronic exposure of pancreatic acinar cells to NNK both *in vitro* (mouse-derived pancreatic acinar 266-6 cells) and *in vivo* (wild-type and transgenic mice that carry the human *SLC19A2* and *SLC19A3* promoters) on thiamin uptake by these cells. The results showed that this compound causes a significant inhibition in thiamin uptake by pancreatic acinar cells and that this inhibition is mediated, at least in part, at the level of transcription of the *SLC19A2* and *SLC19A3* genes.

## Materials and Methods

### Materials

[^3^H]-Thiamin (specific activity 20 Ci/mmol; radiochemical purity >99%) was obtained from American Radiolabeled Chemicals (St. Louis, MO). Nitrocellulose filters (0.45-µm pore size) were from Millipore (Fisher Scientific). Unlabeled thiamin and other chemicals including molecular biology reagents were from commercial vendors and were of analytical grade. Oligonucleotide primers used in this study were synthesized by Sigma Genosys (Sigma, Woodland, TX).

### Cell Culture, Chronic Exposure of 266-6 Cells to NNK, and Uptake Studies

The mouse-derived pancreatic acinar 266-6 cells were from American Type Tissue Collection (ATCC, Rockville, MD) and were cultured in DMEM growth medium containing 10% FBS and an antibiotic cocktail. Cells were used between passages 2 and 20. Chronic exposure of 266-6 cells to 3 µM NNK (a concentration that mimics the level of NNK found in human pancreatic juice of smokers [28]) was done for 24 h in DMEM growth medium containing 5% FBS as described before [33]. Uptake was measured at 37°C in cells suspended in Krebs-Ringer (K–R) buffer (in mM: 133 NaCl, 4.93 KCl, 1.23 MgSO_4_, 0.85 CaCl_2_, 5 glucose, 5 glutamine, 10 HEPES, and 10 MES; pH 7.2) as described before [14]. Labeled (and unlabeled) thiamin was added to the incubation medium at the onset of incubation, and uptake was examined during the initial linear period of uptake (5 min). The reaction was terminated by the addition of 2 ml of ice-cold K–R buffer followed by immediate aspiration. Cells were then rinsed two times with ice-cold K–R buffer, digested with 1 ml of 1 N NaOH, neutralized with 10 N HCl, and then measured for radioactive content using a scintillation counter (Beckman Coulter LS6500, Brea, CA). Digested samples were taken for determining protein concentration (Bio-Rad Dc protein assay kit).

### Chronic Exposure of Mice to NNK, and Thiamine Uptake by Freshly Isolated Pancreatic Acinar Cells

Wild-type and transgenic mice carrying the full-length human *SLC19A2* (-2,250 to −36) and *SLC19A3* (−1,957 to +59) promoters fused to firefly luciferase reporter gene (previously generated and characterized by this lab; 34, 35) were used in these studies. Animal use was approved by the Animal Use Committee of the Veterans Affairs at Long Beach. NNK was given as described recently [26] by intraperitoneal injection of 10 mg/100 g body weight, three times/week for 2 weeks; control transgenic mice were pair-fed and injected with normal saline only (a dosage similar to the level of NNK found in smokers [26]). After 2 week of chronic NNK exposure, mice were euthanized and the pancreas was removed and primary pancreatic acinar cells were isolated from mice by a collagenase type-V (Sigma, St. Louis, MO) digestion method as described previously [36]. After isolation of primary pancreatic acinar cells, they were used fresh for uptake analysis and portion was stored at −80°C for protein, mRNA expression and firefly luciferase analysis. Cells were suspended in K–R buffer and either [^3^H] labeled or unlabeled thiamin was added to initiate uptake. The uptake reaction was terminated by adding 1 ml ice-cold K–R buffer, and the cell suspension was placed on nitrocellulose filters under negative pressure by use of a vacuum manifold (Hoefer Scientific Instruments, Holliston, MA), washed with 5 ml of ice-cold K–R buffer, and dissolved in scintillation fluid. Counting or radioactivity and protein determination were done as described above.

### Western Blot Analysis

Western blot analysis was performed on whole cell lysate prepared from 266-6 cells and from mice primary pancreatic acinar cells chronically exposed to NNK and their respective controls as described previously [14]. The 266-6 cells and mice primary pancreatic acinar cells were suspended in 200 µl of RIPA buffer (Sigma) according to manufacturer’s protocol supplemented with protease inhibitor cocktail (Roche). Equal amounts (60 µg) of pancreatic acinar cell proteins were resolved onto premade 4–12% Bis-Tris minigel (Invitrogen). After electrophoresis, proteins were electroblotted onto immobilon polyvinylidene difluoride membrane (Fisher Scientific) and then blocked with Odyssey blocking solution (LI-COR Bioscience, Lincoln, NE). The membranes were incubated overnight either with mice THTR-1 or THTR-2-specific (1∶200 dilution) polyclonal goat antibodies along with β-actin (1∶3,000 dilution) monoclonal antibody. The THTR-1, THTR-2, and β-actin immunoreactive bands were detected by using donkey anti-goat IRDye-800 and anti-mouse IRDye-680 (LI-COR Bioscience) secondary antibodies (1∶30,000 dilution). Signals were detected and quantitated with the Odyssey infrared imaging system (LI-COR Bioscience) accompanied with LI-COR software for quantification and normalized to β-actin as an internal control.

### Real-Time PCR Analysis

Total RNA (5 µg) was isolated from 266-6 cells (treated with NNK and their controls) and from primary acinar cells of NNK treated mice and their pair-fed controls, then treated with DNase I (Invitrogen) enzyme to prevent genomic DNA contamination. The DNase I-treated samples were subjected to cDNA synthesis using iScript cDNA synthesis kit (Bio-Rad, Hercules, CA). The coding region of mice THTR-1, THTR-2, and ARPO, were PCR amplified using gene-specific primers (Table 1) for quantitative PCR study. Quantitative PCR conditions were same as described previously [14]. The data were normalized to ARPO and then quantified by a relative relationship method [37].

**Table 1:** Primers used for amplifying coding region of the respective genes by quantitative PCR and bisulfite PCR primers for amplifying CpG islands.

Gene Name	Forward and Reverse Primers (5′-3′)
Real time PCR primers	
mTHTR-1	GTTCCTCACGCCCTACCTTC; GCATGAACCACGTCACAATC
mTHTR-2	TCATGCAAACAGCTGAGTTCT; ACTCCGACAGTAGCTGCTCA
mARP0	GCTGAACATCTCCCCCTTCTC; ATATCCTCATCTGATTCCTCC
Bisulfite PCR Primers	
*SLC19A2*	TTGGAGTGGAGTTTTATATGTTTTT; ATACTTACCTACTCTTCCATTAATC
*SLC19A3*	TTGAGGTAGAAGAATTGTTGGAATT; TCACCATATTAACCAAACTCATCTC
*SLC19A3*	GAGATGAGTTTGGTTAATATGGTGAA; TTTTAAAACAAAATCTCCCTCTATC
*SLC19A3*	GTTTTTATATTGGGATGAAAGGTTT; CCATAATAACTTTACCAACAATACC

### Transfection and Reporter Gene Assay

The *SLC19A2* and *SLC19A3* full-length, minimal and the mutated minimal promoter-luciferase reporter constructs utilized in this study were generated previously [34], [35]. 266-6 cells were cotransfected in 12-well plates at less than 80% confluency with 2 µg of each test construct and 100 ng of the *Renilla* transfection control plasmid *Renilla luciferase*-thymidine kinase (pRL-TK) (Promega, Madison, WI). Transfection was performed with lipofectamine 2000 reagent (Invitrogen) according to manufacturer’s instructions. On the second posttransfection day the cells transfected with *SLC19A2* and *SLC19A3* constructs were exposed to NNK (3 µM) for 24 h as described above. At the end of 24 h of NNK exposure, *Renilla*-normalized firefly luciferase activity was determined by using the Dual Luciferase Assay system (Promega).

### Bisulfite Conversion and Sequencing

Methylation status of the *SLC19A2* and *SLC19A3* promoters was assessed by bisulfite sequencing. Full-length promoter–luciferase constructs of *SLC19A2* and *SLC19A3* were transiently transfected in 266-6 cells and were exposed to NNK as described above and DNA was isolated using wizard Genomic DNA purification kit (Promega). The total DNA was digested with *Bam*HI or *Kpn*I for *SLC19A2* and *SLC19A3* respectively. Bisulfite reactions were performed using EpiTect Bisulfite Kit (Qiagen) under conditions that allowed for complete conversion of unmethylated cytosines to uracil, but not 5-methylcytosines. Subsequently, bisulfite-treated DNA was amplified using primers designed by MethPrimer [38] to span areas of CpG islands in the promoter of *SLC19A2* and *SLC19A3*. Primer sequences were designed to contain no CG dinucleotides and are listed in Table 1. The bisulfite-modified DNA was amplified by PCR, using the following conditions: 3 min at 95°C; 40 cycles of 30 sec at 95°C, 30 sec at 55°C, and 30 sec at 72°C; and finally 20 min at 72°C. Amplified products were cloned into the pGEM-T easy vector via TA cloning (Promega). At least ten clones were sequenced (per target promoter). The methylation profile of the promoter of interest was determined by comparing the sequence of bisulfite-converted DNA with that of unmodified DNA.

### Statistical Analysis

Uptake data with mouse primary pancreatic acinar cells presented in this paper are mean ± SE of at least three separate experiments from different mice and are expressed as percentage relative to simultaneously performed controls. Uptake with the 266-6 cells is also mean ± SE from at least three separate experiments. Carrier-mediated thiamin uptake was determined by subtracting uptake by simple diffusion from total uptake. Protein, RNA and luciferase activity determinations were performed from at least three sets of samples prepared at different occasions. The Student’s *t*-test was used for statistical analysis, and *P*<0.05 was considered statistically significant.

## Results

### Effect of Chronic Exposure of Pancreatic Acinar Cells to NNK on Thiamin Uptake: *in vitro* Studies

**Figure 1:**
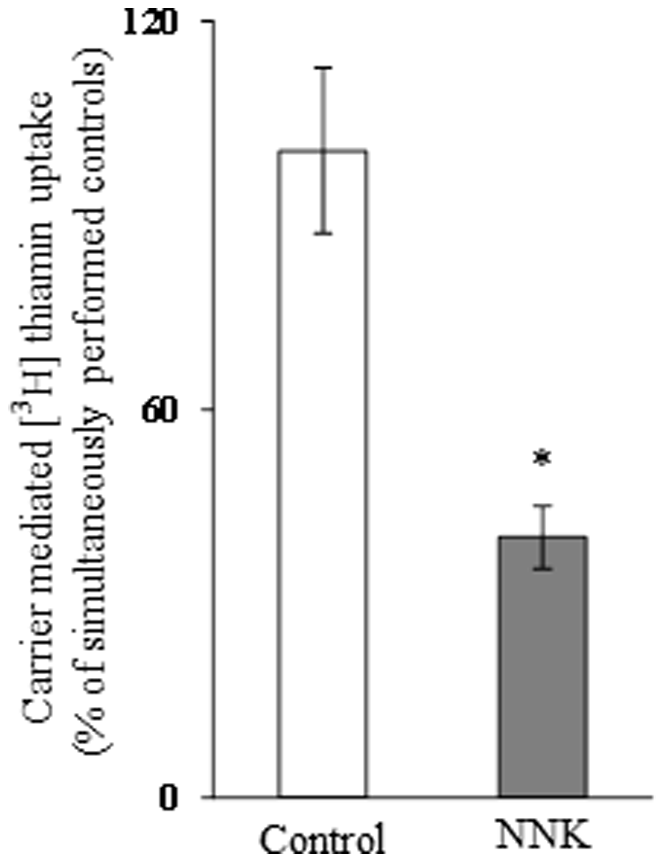
Effect of chronic exposure of pancreatic acinar 266-6 cells to NNK on carrier-mediated [^3^H] thiamin uptake. The initial rate of carrier-mediated uptake of a physiological concentration of [^3^H]thiamin (15 nM) by 266-6 cells exposed to NNK (3µM, 24 h) was determined as described under “Materials and Methods”. Each data represents the mean ± SE of at least three separate uptake determinations. *P<0.01.

**Figure 2:**
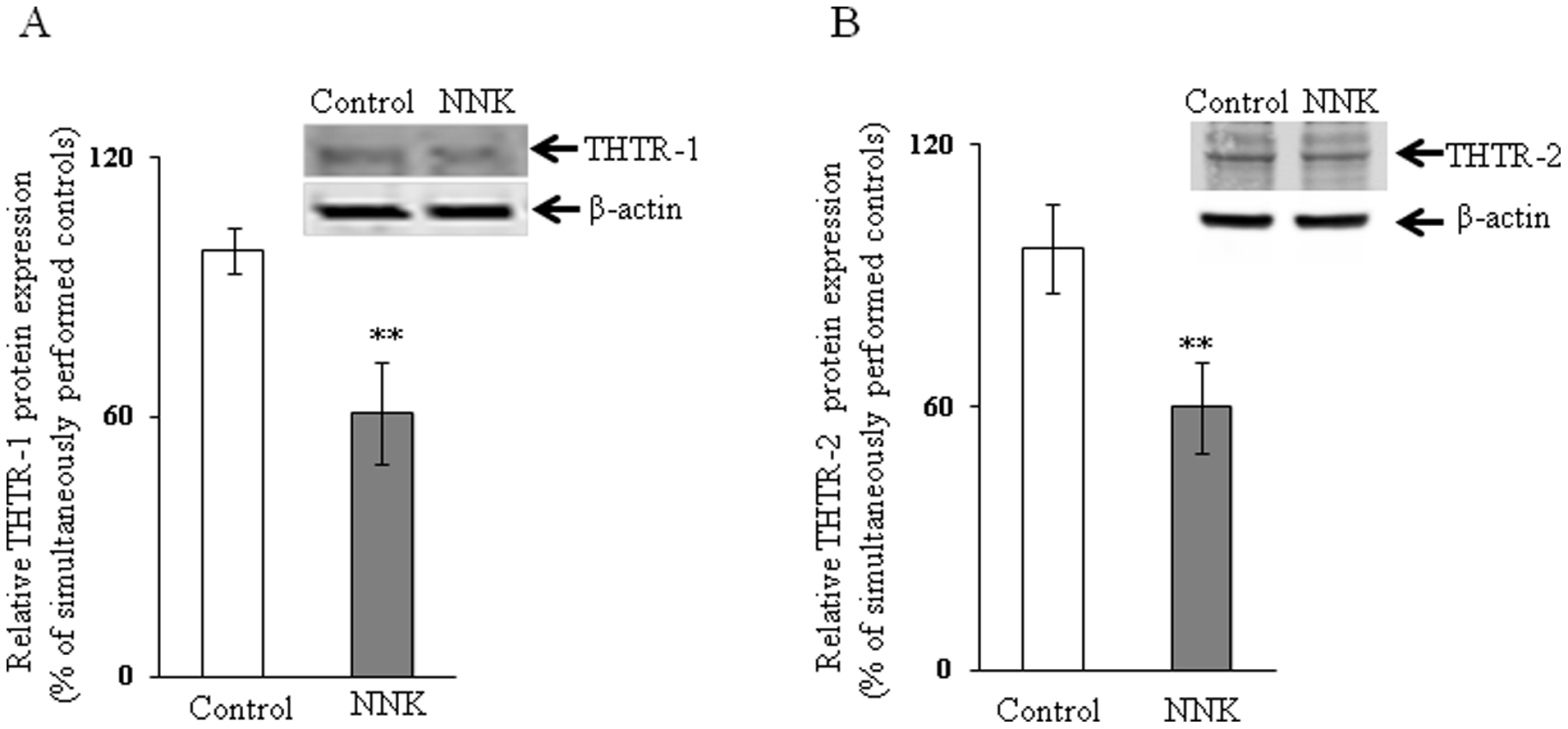
Effect of chronic exposure of pancreatic acinar 266-6 cells to NNK on the level of expression of THTR-1 and THTR-2 proteins. Western blot was performed on whole cell proteins (60 µg) isolated from NNK exposed and control 266-6 cells as described under “Materials and Methods”. A and B: blots were incubated with goat polyclonal THTR-1 and THTR-2 antibody respectively. Data are mean ± SE of at least 3 independent experiments and were normalized relative to β-actin protein expression. **P<0.05.

We examined the effect of chronic exposure of the mouse-derived pancreatic acinar 266-6 cells to NNK (3 µM, for 24 h; 33) on the initial rate of thiamin (15 nM) uptake. The result showed a significant inhibition (*P<*0.01) in carrier-mediated thiamin uptake by cells chronically treated with NNK compared to controls (Figure 1). We also examined the effect of chronic exposure of 266-6 cells to NNK on the level of expression of the mouse THTR-1 and THTR-2 proteins. This was done by means of Western blot analysis using specific polyclonal antibodies against these transporters. The results showed a significant (*P<*0.05 for both) reduction in the level of expression of THTR-1 and THTR-2 proteins in cells chronically exposed to NNK compared to controls (Figure 2).

**Figure 3:**
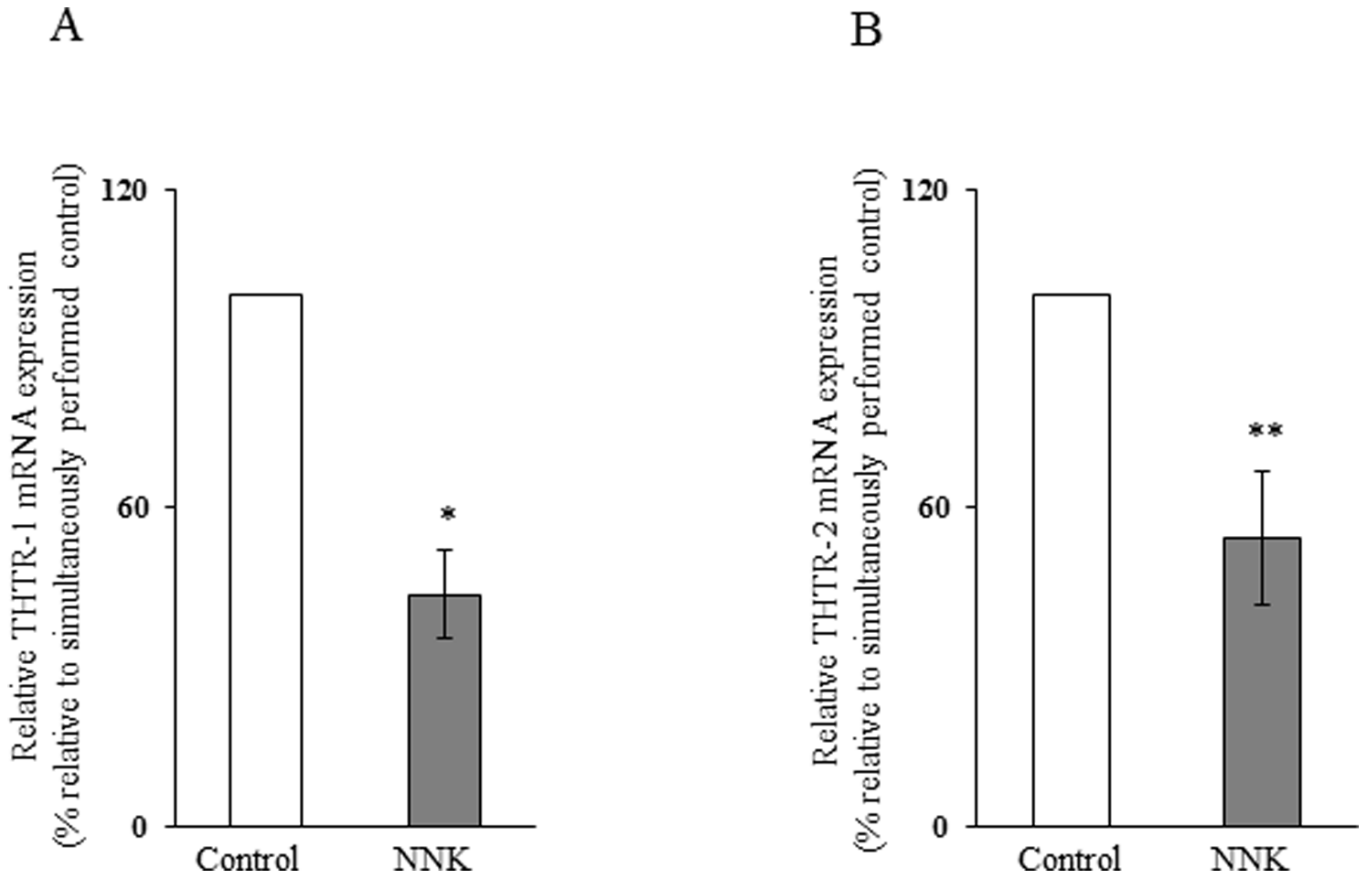
Effect of chronic exposure of pancreatic acinar 266-6 cells to NNK on the level of expression of THTR-1 and THTR-2 mRNA. Q PCR was performed using mice THTR-1 (A) and THTR-2 (B) gene-specific primers and cDNA prepared from NNK exposed as well as control 266-6 cells RNA. Data are mean ± SE of at least 3 independent experiments and were normalized relative to ARPO and calculated by the relative relationship method. *P<0.01, **P<0.05.

In another study, we tested the effect of chronic exposure of pancreatic acinar 266-6 cells to NNK on the level of expression of THTR-1 and THTR-2 mRNA. This was done by quantitative PCR using specific primers for these transporters. The results showed a significant (*P<*0.01 for THTR-1 and *P*<0.05 for THTR-2) reduction in the level of expression of THTR-1 and THTR-2 mRNA in 266-6 cells chronically exposed to NNK compared to controls (Figure 3).

**Figure 4:**
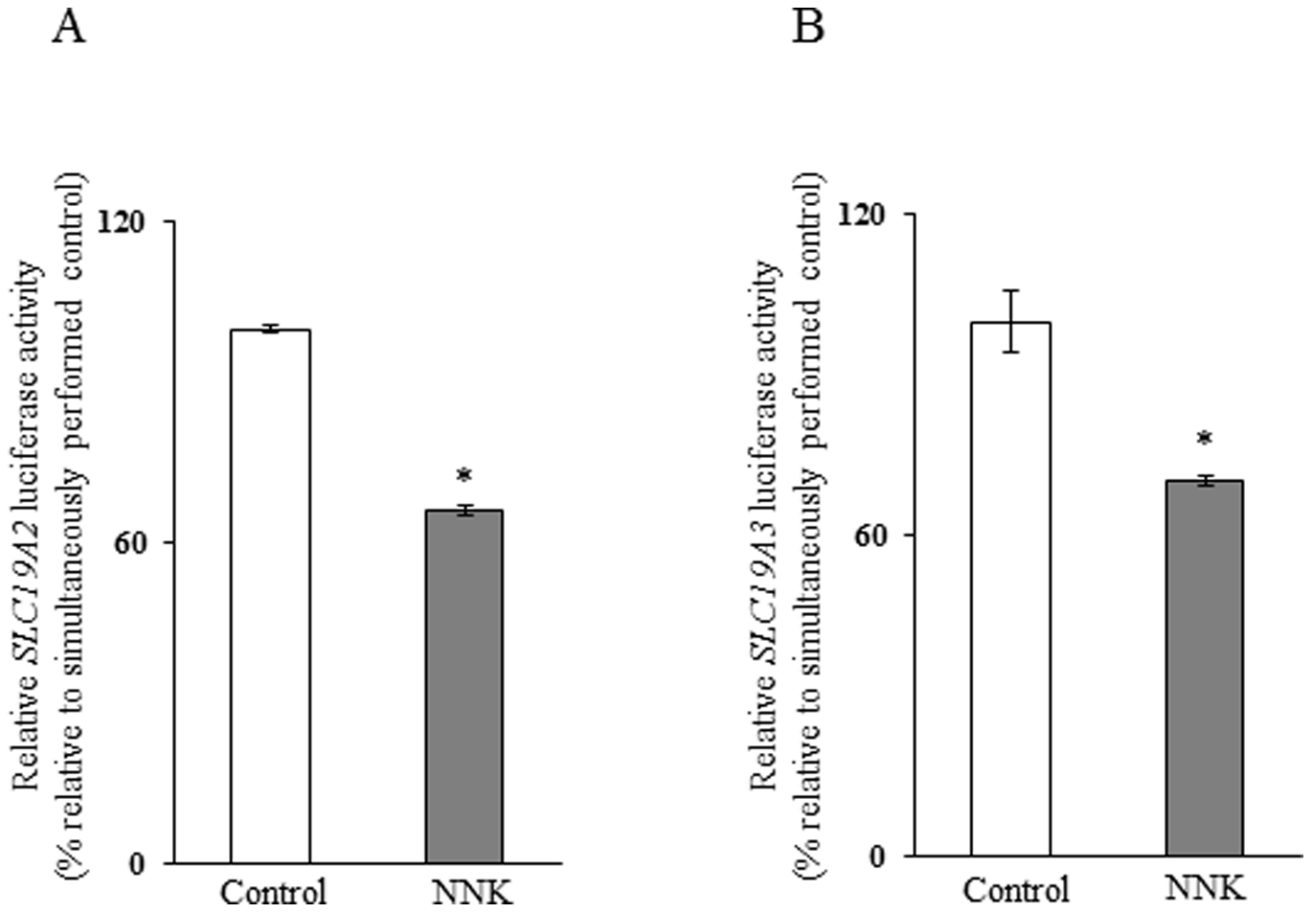
Effect of chronic exposure of pancreatic acinar 266-6 cells to NNK on activity of *SLC19A2* and *SLC19A3* promoters. Full-length *SLC19A2* (A) and *SLC19A3* (B) promoters in pGL3-Basic were transfected into 266-6 cells were exposed to 3 µM NNK for 24 h followed by determination of luciferase activity. Data were normalized relative to *Renilla* luciferase activity and presented as mean ± SE of at least 3 independent experiments. *P<0.01.

While changes in mRNA level could be mediated via different mechanisms, a common mechanism is by changes in the transcription rate of the relevant gene. Thus, we tested the effect of chronic exposure of pancreatic acinar 266-6 cells to NNK on the activity of the *SLC19A2* and *SLC19A3* promoters transfected into these cells. The results showed a significant (*P*<0.01 for both) reduction in the activity of both *SLC19A2* and *SLC19A3* promoters as a result of exposure to NNK (Figure 4). These findings suggest that the effect of chronic exposure to NNK on thiamin uptake by pancreatic acinar cells is exerted, at least in part, at the level of transcription of the *SLC19A2* or *SLC19A3* genes.

### Effect of Chronic Exposure of Mice to NNK on Thiamin Uptake by Pancreatic Acinar Cells: *in vivo* Studies

**Figure 5:**
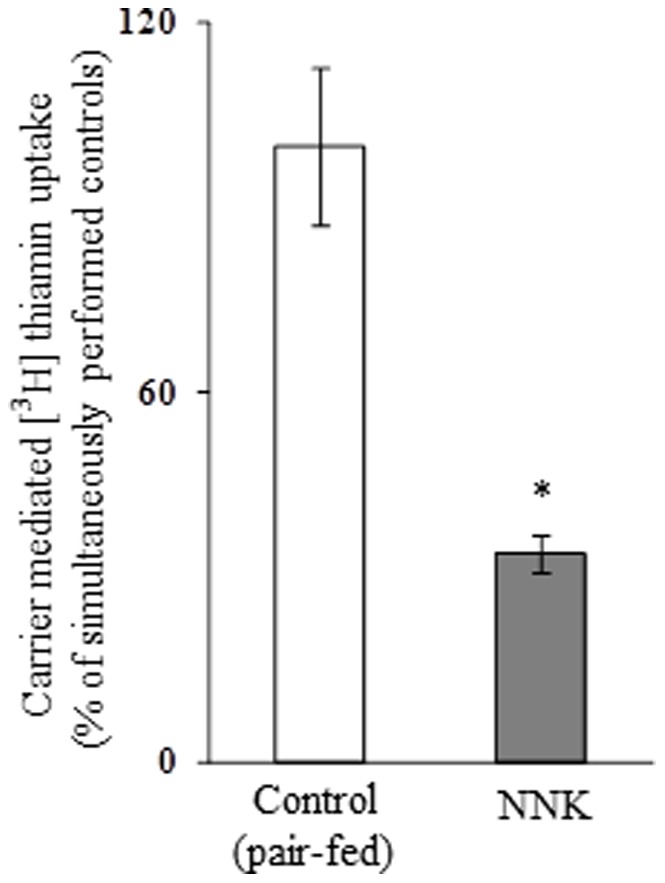
Effect of chronic exposure of mice to NNK on carrier-mediated [^3^H]-thiamin uptake by primary pancreatic acinar cells. Primary pancreatic acinar cells were isolated from mice exposed to NNK (10 mg/100 g body weight, 26) and from their pair-fed controls. Carrier-mediated thiamin uptake was determined as described in Methods. Data are mean ± SE of at least 3 separate uptake determinations from multiple sets of mice. *P<0.01.

**Figure 6:**
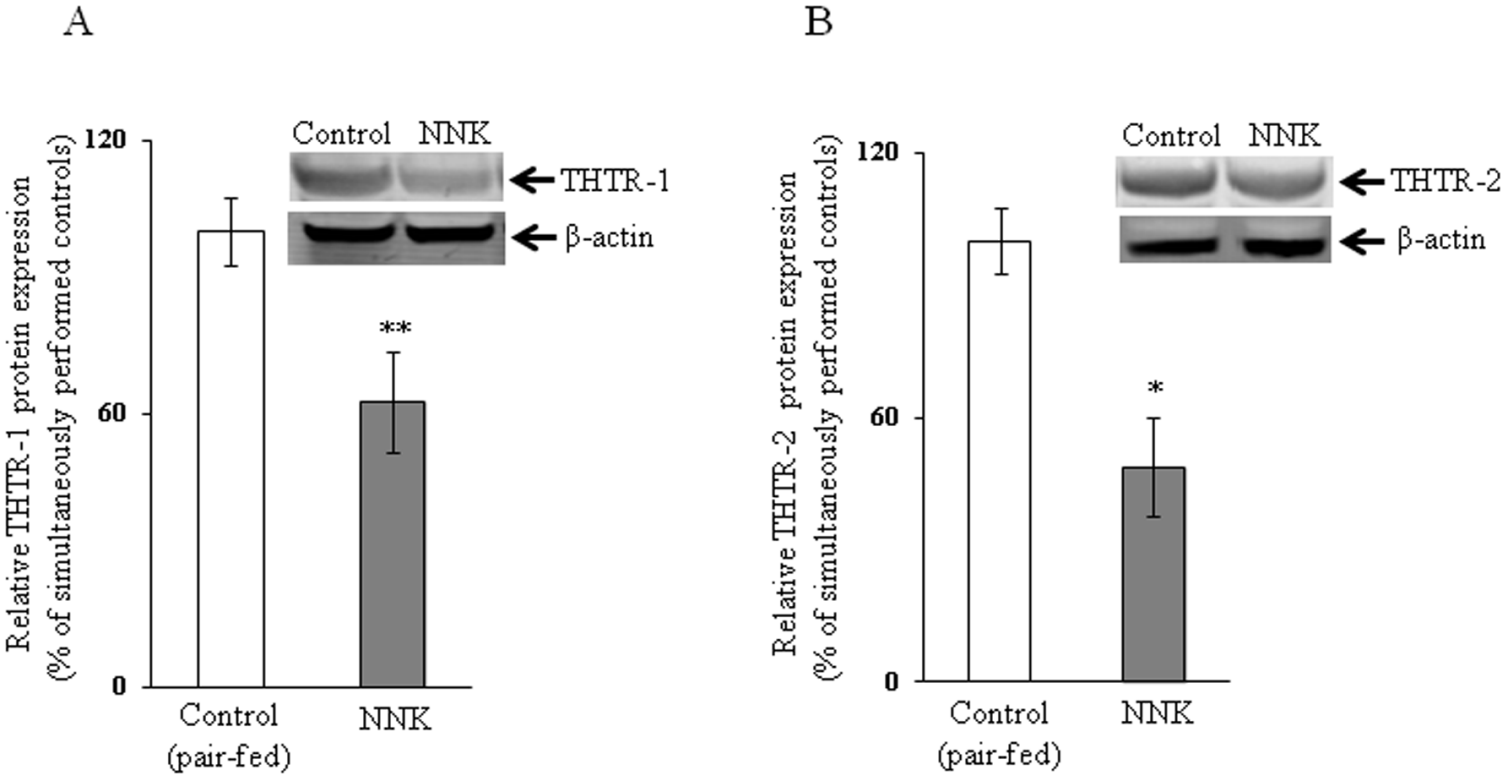
Effect of chronic exposure of mice to NNK on the level of expression of THTR-1 and THTR-2 proteins in primary pancreatic acinar cells. Western blot analysis was done using pancreatic acinar whole cell proteins (60 µg) isolated from chronic NNK exposed mice and their pair-fed controls. A and B: blots were incubated with goat polyclonal THTR-1 and THTR-2 antibody respectively. Data are mean ± SE of at least 3 independent experiments and were normalized relative to β-actin protein expression. *P<0.01, **P<0.05.

To confirm the above-described *in vitro* findings of the effect of chronic exposure of pancreatic acinar cells to NNK on physiological and molecular parameters of thiamin uptake by pancreatic acinar cells *in vivo*, we examined the effect of chronic exposure of mice to NNK on thiamin uptake by pancreatic acinar cells. NNK was administered via IP injection at a dose of 10 mg/100 g body weight, three times per week for 2 weeks as described previously [26]; thiamin uptake studies were done on freshly isolated pancreatic acinar cells. Control mice were injected with saline and were pair-fed. The results showed a significant (*P<*0.01) inhibition in the initial rate of thiamin uptake by pancreatic acinar cells from the NNK-treated mice compared to those from pair-fed controls (Figure 5). We also examined, by means of Western blotting, the effect of chronic exposure of mice to NNK on the level of expression of the mouse THTR-1 and THTR-2 proteins. The results showed a significant (*P<*0.05 for THTR-1 and 0.01 for THTR-2) reduction in the level of expression of THTR-1 and THTR-2 proteins in pancreatic acinar cells of mice chronically exposed to NNK compared to pair-fed controls (Figure 6).

**Figure 7:**
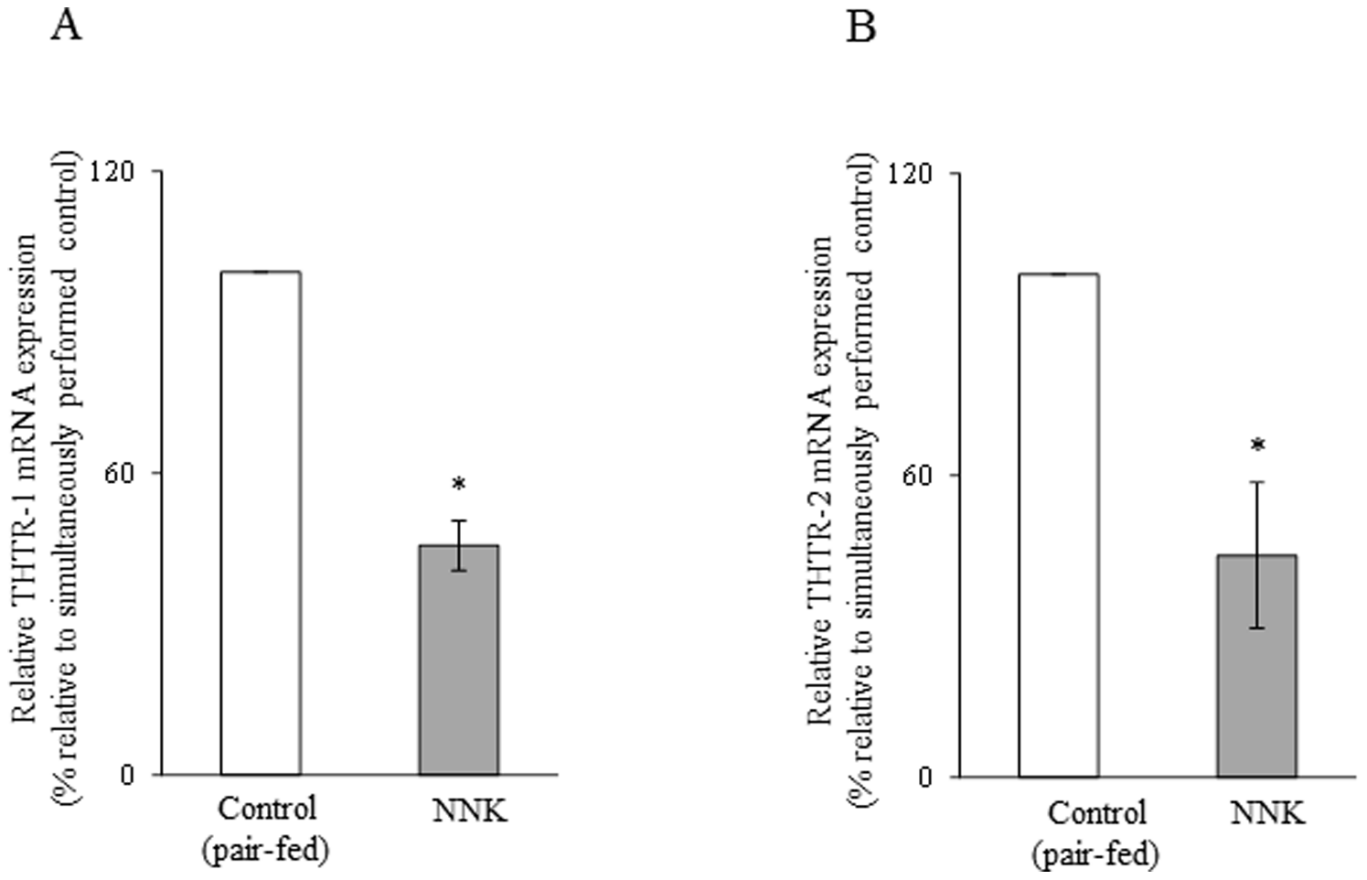
Effect of chronic exposure of mice to NNK on the level of expression of THTR-1 and THTR-2 mRNA in pancreatic acinar cells. QPCR was performed using mice THTR-1 (A) and THTR-2 (B) gene-specific primers and cDNA prepared from pancreatic acinar RNA of NNK-treated mice and their pair-fed controls. Data are mean ± SE from separate sets of samples from multiple mice and were normalized relative to ARPO and calculated by the relative relationship. *P<0.01.

In another study, we examined (by means of quantitative PCR) the effect of chronic exposure of mice to NNK on the level of expression of THTR-1 and THTR-2 mRNA. The results showed a significant (*P<*0.01 for both) reduction of expression of the THTR-1 and THTR-2 messages in pancreatic acinar cells of mice chronically treated with NNK compared to pair-fed controls (Figure 7).

**Figure 8:**
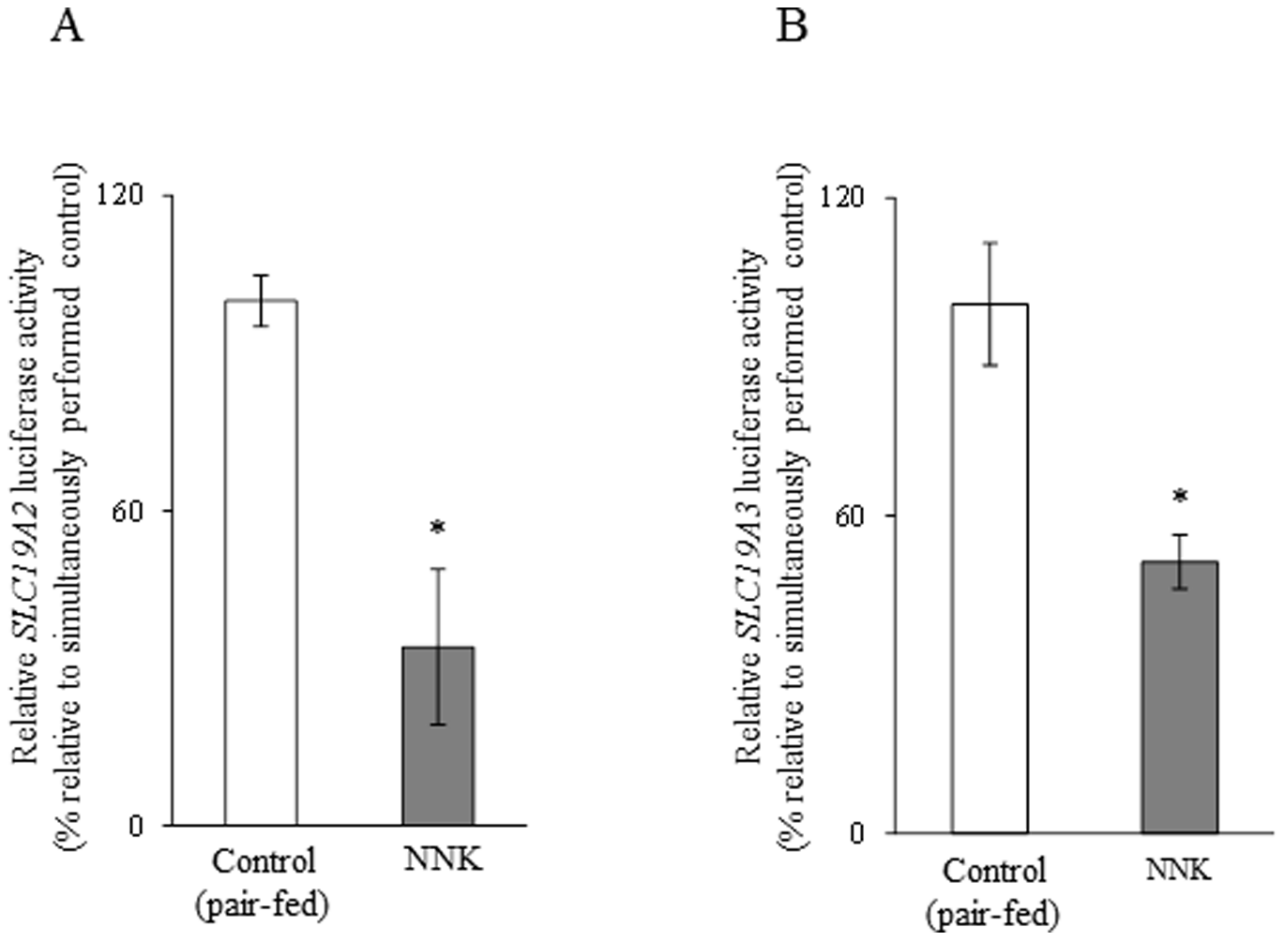
Effect of chronic exposure of transgenic mice carrying *SLC19A2* and *SLC19A3* promoters to NNK on the activity of these promoters. The activity of *SLC19A2* (A) and *SLC19A3* (B) promoters were determined as described in “Materials and Methods” and are presented as percentage relative to pair-fed controls. Data are mean ± SE of at least 3 independent experiments from multiple sets of mice. *P<0.01.

We also examined the effect of chronic exposure of mice to NNK on activity of the *SLC19A2* and *SLC19A3* promoters *in vivo*. For this, we used our previously established and well- characterized mice lines that carry the human *SLC19A2* and *SLC1A3* promoters fused to the luciferase reporter gene [34], [35]. Treatment with NNK was done as described earlier and pair feeding of the control transgenic mice was instituted. The results showed a significantly (*P<*0.01 for both) lower activity of the *SLC19A2* and *SLC19A3* promoters in pancreatic acinar cells of transgenic mice chronically exposed to NNK compared to activity of transgenic controls (Figure 8).

### Effect of Chronic Exposure of Pancreatic Acinar Cells to NNK on DNA Methylation Profile of the *SLC19A2* and *SLC19A3* Promoters

One of the mechanisms through which a given condition affects transcriptional activity of a particular gene is via epigenetic mechanisms like DNA methylation (i. e., via changing the methylation status of CpG islands of the gene promoters; 39). In general, hypo-methylation of the DNA surrounding a gene promoter is believed to activate the gene, while hyper-methylation silences the gene [39]. Since NNK (and CS) is known to affect DNA methylation of many other genes [33], [40] we investigated whether chronic treatment of pancreatic acinar 266-6 cells with this CS component also affected the methylation status of the *SLC19A2* and *SLC19A3* promoters. We used Methprimer [38] to screen for CpG islands in the *SLC19A2* and *SLC19A3* full-length promoter region. The default parameters for defining CpG islands were, 1) an observed/expected CpG ratio over 0.6, 2) the percentage of G plus C over 50%, and 3) a window size of at least 100 bp. Our Methprimer analysis identified one CpG island of 536 bp for *SLC19A2* and three CpG island of 112, 108 and 114 bp for *SLC19A3*. We then used the results of the Methprimer analysis to design primers that did not contain CpG dinucleotide sequences (Table 1) and amplified the CpG regions of the respective promoters after bisulfite conversion of DNA isolated from control and NNK exposed 266-6 cells followed by sequencing for the identified regions as described previously [41]. The results, however showed, no significant alterations in DNA methylation at the CpG sites of the *SLC19A2* and *SLC19A3* promoters as a result of chronic exposure to NNK. These findings suggest that other mechanism(s) are involved in mediating the inhibitory effects of chronic exposure to NNK on activity of the *SLC19A2* and *SLC19A3* promoters.

### Role of the Minimal Regions of the *SLC19A2* and *SLC19A3* Promoters in Mediating the NNK Effect, and Identification of the Cis-elements Involved

**Figure 9:**
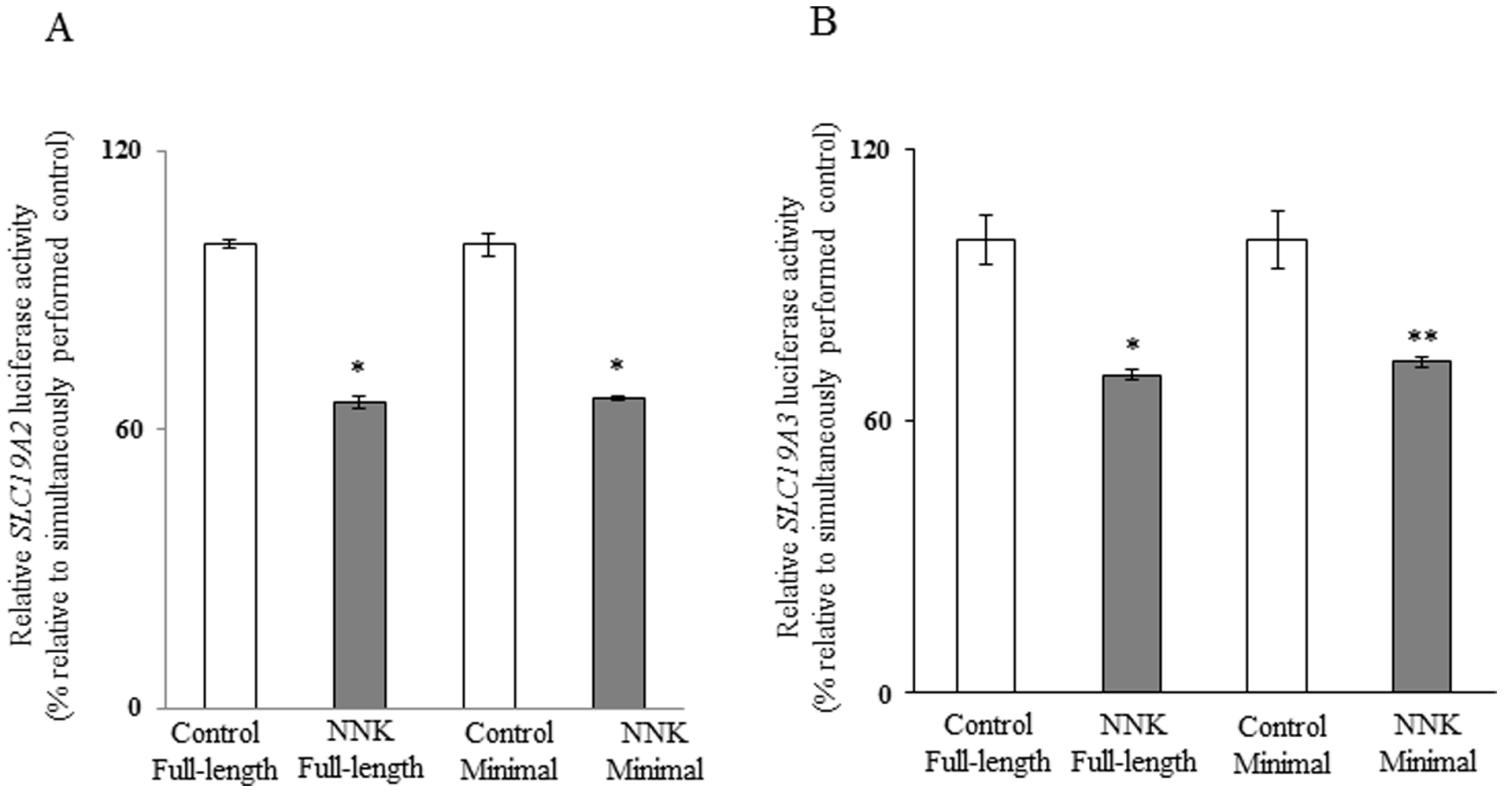
Role of the minimal regions of the *SLC19A2* and *SLC19A3* promoters in mediating the NNK effect. Full-length and minimal *SLC19A2* (A) and *SLC19A3* (B) promoters in pGL3-Basic were transiently transfected in 266-6 pancreatic acinar cells; luciferase assays were performed as described under “Materials and Methods”. Data are reported as relative firefly luciferase activity normalized to *Renilla* luciferase activity and represent mean ± SE of at least three independent experiments. *P<0.01, **P<0.05.

**Figure 10:**
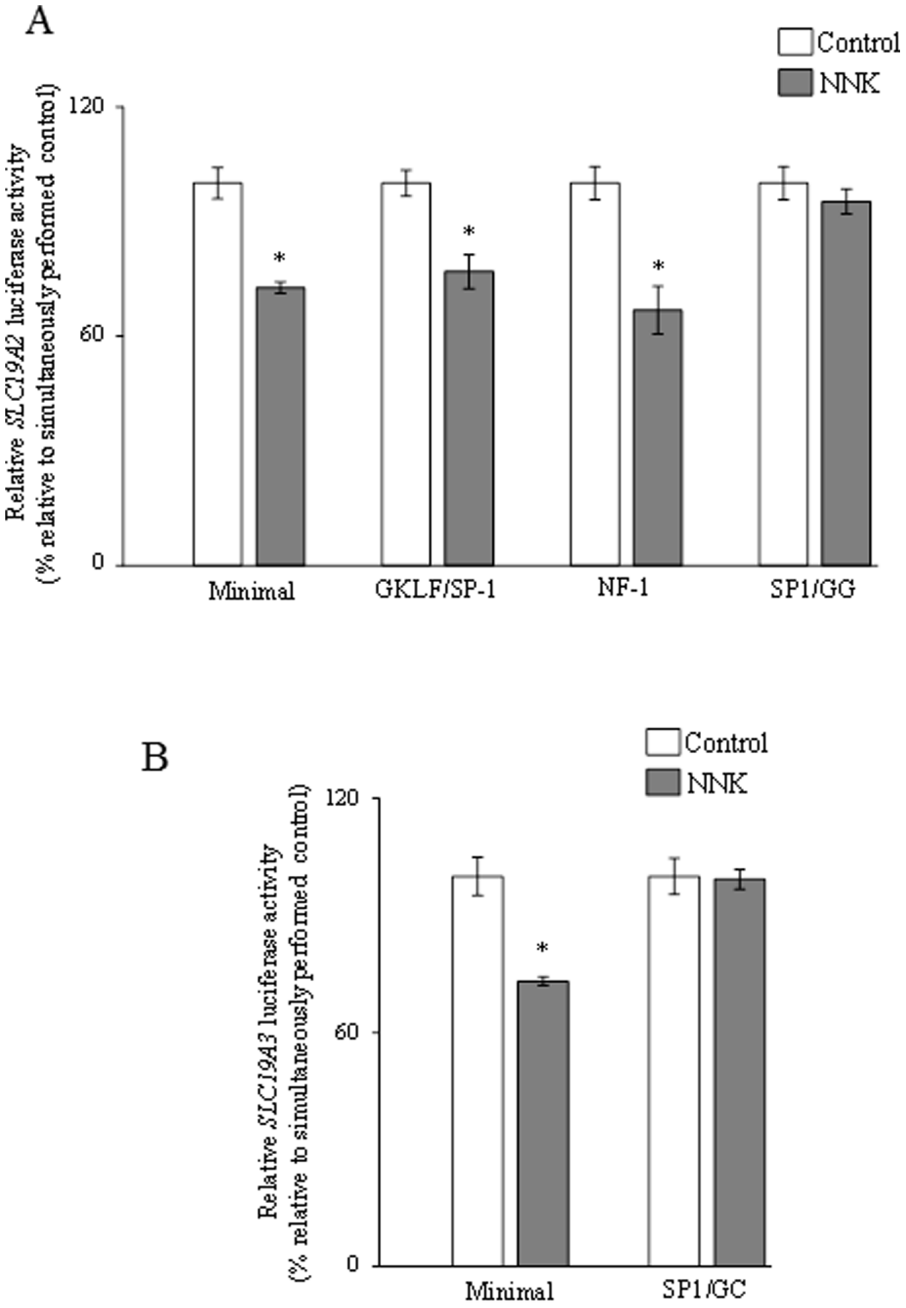
Role of specific *cis*-regulatory elements in the minimal region of the *SLC19A2* and *SLC19A3* promoters in mediating the effect of chronic exposure to NNK. The mutated NNK-responsive minimal region of the *SLC19A2* (*A*) and *SLC19A3* (*B*) promoters in pGL3-Basic were transiently transfected into 266-6 cells and the luciferase assays was determined. Data are reported as relative firefly luciferase activity normalized to *Renilla* luciferase activity and represent mean ± SE of at least three independent experiments. *P<0.01.

Our aim in this study was to determine if the previously identified [34], [35] minimal regions required for basal activity of the *SLC19A2* and *SLC19A3* promoters are involved in mediating the NNK effect on activity of these promoters. Thus, we examined and compared the effect of chronic exposure to NNK on the activity of the full-length vs the minimal *SLC19A2* and *SLC19A3* promoters in pancreatic acinar 266-6 cells. The results showed NNK to cause a similar degree of inhibition in activity of the full-length and the minimal *SLC19A2* and *SLC19A3* promoters (Figure 9), suggesting that the minimal region is involved in mediating this inhibition. Previous studies from our laboratory have shown that the minimal promoter regions of the *SLC19A2* and *SLC19A3* promoters contain cis-regulatory elements that are important for their activity [34], [35]. For the *SLC19A2* promoter, the cis-regulatory elements GKLF/SP-1, NF-1 and SP-1/GG box binding sites were found (by means of mutational analysis) to be important for its activity [34]; For the *SLC19A3* promoter, the cis regulatory element SP-1/GC-box binding site was found to be important for its activity [35]. Thus, we examined the effect of mutations at these sites on the inhibitory effect of NNK on the activity of the minimal *SLC19A2* and *SLC19A3* promoters. The results showed that while mutating the GKLF/SP-1 and NF-1 binding sites did not interfere with the inhibitory effect of NNK on activity of the *SLC19A2* minimal promoter, mutating SP-1/GG-box site lead to a complete disappearance of the inhibitory effect (Figure 10 A). Similarly, mutating the SP-1/GC-box lead to a complete disappearance of the NNK inhibitory effect on activity of the *SLC19A3* promoter (Figure 10 B).

## Discussion

As mentioned earlier, thiamin is important for normal function of pancreatic acinar cells and these cells obtain the vitamin from their surrounding via a specific carrier-mediated process that involves both the plasma membrane THTR-1 and THTR-2 [15]. Also, there is a wealth of evidence to suggest that cigarette smoking is a significant risk factor for the development and progression of pancreatic injury [16]–[25]. Furthermore, components of CS, e.g., NNK, exert multiple negative effects on pancreatic acinar physiology and function [23], [26]. Nothing, however, is known about possible effect of NNK (or any other component of CS) on the physiology of pancreatic acinar uptake of vitamin B1. Addressing this issue is of physiological and nutritional importance as impairment in normal cellular thiamin physiology could negatively impact the resting state of the exocrine pancreas and to lowering of its defense mechanisms leading to an increase in its susceptibility to injury. Therefore, our aim in this study was to investigate the effect of chronic exposure of pancreatic acinar cells to NNK on physiological and molecular parameters of the pancreatic acinar thiamin uptake. We examined these issues using mouse-derived pancreatic acinar 266-6 cells as an *in vitro* mode of exposure, and wild-type and transgenic mice carrying the human *SLC19A2* and *SLC19A3* promoters as *in vivo* models. The results showed that chronic exposure of 266-6 cells to NNK lead to a significant inhibition in carrier-mediated thiamin uptake. The inhibition was associated with a significant reduction in the expression of THTR-1 and THTR-2 at the protein and mRNA levels. While changes in mRNA levels could occur via different mechanisms, one such mechanism is changes in the transcription rate. The latter appears to be the case here as significant reduction in the activity of the *SLC19A2* and *SLC19A3* promoters transfected into 266-6 cells was observed upon chronic exposure to NNK.

To confirm and extend the *in vitro* findings to the *in vivo* situation, we then chronically exposed mice to NNK and examined the effect of that exposure on physiological and molecular parameters of pancreatic acinar thiamin uptake. The results again showed a significant inhibition in pancreatic acinar thiamin uptake, and this inhibition was associated with a significant reduction in the expression of mouse THTR-1 and THTR-2 at the protein and mRNA levels. Involvement of transcriptional mechanism in the latter effect was confirmed in studies using transgenic mice carrying the *SLC19A2* and *SLC19A3* promoters and chronically exposed to NNK. In the latter studies, a significant reduction in the activity of the *SLC19A2* and *SLC19A3* promoters in pancreatic acinar cells of the transgenic mice exposed to NNK as compared to their activity in pair-fed transgenic animals was observed. Results of our complementary *in vitro* and *in vivo* approaches suggest that NNK inhibits pancreatic acinar thiamin uptake and that at least part of this effect is exerted at the level of transcription of the *SLC19A2* and *SLC19A3* genes.

It is well established that gene transcription can be modulated by epigenetic mechanisms like DNA methylation [39]. Since exposure to NNK (and to CS) is known to cause changes in DNA methylation of certain genes [33], [40], we sought to determine if this was the case with the *SLC19A2* and *SLC19A3* promoters. Thus, we investigated the effect of chronic exposure to NNK on methylation profile of the *SLC19A2* and *SLC19A3* promoters. DNA methylation usually involves CpG islands, and thus, we performed a Methprimer [38] analysis to identify the CpG islands in the *SLC19A2* and *SLC19A3* promoters, followed by bisulfite sequencing [41]. The results, however, showed no significant changes in the methylation profile of the *SLC19A2* and *SLC19A3* promoters as a result of chronic exposure to NNK, suggesting that other mechanism(s) are involved in mediating the inhibitory effects of NNK on activity of the *SLC19A2* and *SLC19A3* promoters. To shed light on this other mechanism(s), we determined if the minimal regions of the *SLC19A2* and *SLC19A3* promoters needed for basal activity of the respective gene are involved in mediating can also mediate the NNK inhibitory effect. For this, we examined and compared the effect of chronic exposure to NNK on the activity of the minimal and full-length *SLC19A2* and *SLC19A3* promoters expressed in 266-6 cells and found a similar degree of inhibition with both constructs, suggesting involvement of the minimal regions in mediating the NNK inhibitory effect. Knowing that the minimal region of the *SLC19A2* promoter contains the cis-elements GKLF/SP-1, NF-1 and SP-1/GG box binding sites and that these elements are important for its function [34], we examined the effect of mutating these sites on the NNK effect on *SLC19A2* promoter activity. The results showed the disappearance of the inhibitory effect of NNK on the *SLC19A2* promoter only when the SP-1/GG-box site was mutated, suggesting involvement of this site in mediating the NNK effect. Similarly the *SLC19A3* minimal promoter is known to have an SP-1(SP-3)/GC-box binding site that is important for its activity [35]. Mutating this site again led to disappearance of the inhibitory effect of NNK on *SLC19A3* promoter activity, which suggest involvement of the site in mediating the NNK effect.

In conclusion, this study demonstrates for the first time that chronic exposure of pancreatic acinar cells to NNK negatively impacts the physiological and molecular parameters of thiamin uptake by these cells and this effect is exerted, at least in part, at the level of transcription of the *SLC19A2* and *SLC19A3* genes.
